# Impact of the COVID-19 Pandemic on Burnout in Primary Care Physicians in Catalonia

**DOI:** 10.3390/ijerph18179031

**Published:** 2021-08-27

**Authors:** Gemma Seda-Gombau, Juan José Montero-Alía, Eduard Moreno-Gabriel, Pere Torán-Monserrat

**Affiliations:** 1 Unitat de Suport a la Recerca Metropolitana Nord, Institut Universitari d’Investigació en Atenció Primària Jordi Gol (IDIAP Jordi Gol), 08303 Mataró, Spain; jjmontero.bnm.ics@gencat.cat (J.J.M.-A.); emoreno@idiapjgol.info (E.M.-G.); ptoran.bnm.ics@gencat.cat (P.T.-M.); 2 Departament d’Empresa, Facultat d’Economia i Empresa, Universitat Autònoma de Barcelona, 08193 Bellaterra (Cerdanyola del Vallès), Spain; 3 Multidisciplinary Research Group on Health and Society (GREMSAS), (2017 SGR 917), 08007 Barcelona, Spain; 4 Centre d’Atenció Primària Mataró-3 (Rocafonda-Palau), Gerència Àmbit Metropolitana Nord, Institut Català de la Salud, 08304 Mataró, Spain; 5 Department de Medicina, Facultat de Medicina, Universitat de Girona, 17004 Girona, Spain

**Keywords:** burnout, primary care, physicians, COVID-19, Catalonia, Spain

## Abstract

Background: Recent demands to raise the clinical quality, improve the patient experience, and decrease costs have progressively increased burnout among primary care physicians. This overstretched situation has been greatly aggravated since the onset of the COVID-19 pandemic. The aim of the study is to analyse the prevalence of burnout among primary care physicians and to assess the impact of the COVID-19 pandemic on burnout. Methods: This was a multicentre longitudinal descriptive study of occupational factors and burnout before and since the start of the COVID-19 pandemic. In order to assess the impact of the pandemic on burnout in primary care physicians, two paired groups of physicians were compared using Wilcoxon’s and McNemar’s tests. Results: In January 2019, 10% of primary care physicians scored high on all burnout domains. Seven months into the COVID-19 pandemic (October 2020), this percentage increased to 50%. Paired groups analysis showed unprecedented worsening due to the pandemic: emotional exhaustion, which already affected 55% of primary care physicians, jumped to 77%. Conclusions: Burnout is endemic among primary care physicians. It has been associated with lower patient satisfaction, reduced health outcomes, and increased costs. The COVID-19 pandemic has pushed burnout in primary care professionals to the edge.

## 1. Introduction

The clinical syndrome of occupational burnout was firstly described in 1974 by Herbert Freudenberger in New York [1]. It was defined in 1981 by Maslach and Jackson [2] as “a syndrome of emotional exhaustion, depersonalization and low personal fulfilment, which might occur in public-facing professionals.” Burnout is an important psychosocial occupational disease, which has been associated with physical, behavioural, and emotional distress. While it does not usually incapacitate the professional, it impairs the quality of care and the relationship with colleagues [3]. In general, physicians have a higher prevalence of burnout than most occupations, and rates of burnout are higher in family doctors than amongst hospital specialists [3,4,5].

Burnout has several conceptualizations and explanatory models [6]. However, they all involve the perception that demand outweighs resources in occupational settings [3]. Recent pressures to decrease the cost of healthcare, raise clinical quality, and improve patient experience have overburdened clinicians [3]. An emotionally demanding job with insufficient resources and chronic stress makes health workers particularly vulnerable to burnout. A large number of general practitioners are affected by high workload, patient complexity, short times allocated per visit and a culture of blame and intimidation that compromise the values of family and community medicine. Medical errors, absenteeism, and lower productivity are higher in physicians affected by burnout. Undoubtedly, physician burnout affects human health and negatively impacts the economy of health institutions [7,8,9,10].

Over the past few years, a large number of doctors across Europe have been on the brink of exhaustion, of deterioration of their own health, and considering abandoning their profession [6]. In a study conducted in over 12,000 physicians in 2015, approximately 30% of family doctors in Britain anticipated leaving clinical practice within 5 years [10].

Various evidence-based individual and organizational strategies have been used to decrease burnout. For instance, studies have found that mindfulness, stress management, and communication skills training, exercise and self-care programs, small group participation and discussion can reduce physician exhaustion. Changes in the work environment driven by the organization can also significantly reduce professional exhaustion [11,12,13]. The COVID-19 pandemic has significantly disrupted the work environment of healthcare professionals across the globe, changing the factors affecting burnout and the initiatives aimed at reducing it. Healthcare workers have suffered an unprecedented increase in workload with limited resources, further impairing their coping mechanisms. Eighteen months into the pandemic, the literature already reports high levels of burnout and associated mental disorders in this population. In hospital staff, the pandemic has increased depression, anxiety, and insomnia [14]. Using the Maslach Burnout Inventory (MBI) in Italian frontline healthcare workers, a study reports that over 33% showed high scores for emotional exhaustion and 25% high levels of depersonalization. In contrast, only 15% reported low levels of personal gratification [15]. In Spain, Luceño-Moreno et al. [16] have explored the relationship between burnout and various mental health issues in health professionals during COVID-19.

The evolution of burnout in primary care physicians in the Barcelona Metropolitan Area has been scarcely explored. A longitudinal study published in 2008 showing an increase in burnout after 5 years of follow up stressed the negative impact of adverse working conditions [14].

In 2016, the Catalan Society of Family and Community Medicine (CAMFiC) and the Maresme Regional Board of the College of Physicians of Barcelona (COMB) sponsored the launch of the Observatory of Family and Community Medicine, with the aim to monitor the workload of primary care physicians over time. For this study, primary care physicians from the Maresme region (northern metropolitan area of Barcelona) were invited in 2016, 2019, and 2020 to respond to various surveys that included socio-demographic data, daily working hours, and occupational well-being.

Drawing on the data collected from this study, this paper not only presents burnout prevalence and evolution among primary care physicians in the Barcelona metropolitan area during the past 5 years, but measures the effect of the COVID-19 pandemic on this pre-existing burnout.

The study hypothesis is that the COVID-19 crisis has greatly worsened burnout in all three dimensions (emotional exhaustion, depersonalization, and personal accomplishment) among primary care physicians. 

## 2. Materials and Methods

This is a multi-centre descriptive study using a convenience sampling on the workload of primary care physicians and how this workload affects them emotionally and professionally. The area of the study consists of 30 towns with an accumulation of over 400,000 inhabitants, 21 primary care teams, and approximately 260 primary care physicians. The survey was conducted in November 2016 (T1), January 2019 (T2), and October 2020 (T3), with the participation of 150 professionals (40 have responded to the 3 questionnaires). In this study, we focus only on the 40 respondents to the three questionnaires, with the aim to describe the evolution of the emotional impact over time.

### 2.1. Instrument

Burnout was measured using the MBI for medical professionals [17]. The MBI is a psychological assessment instrument comprising 22 items related to occupational burnout and takes about 10 min to complete. The MBI measures three dimensions of burnout: emotional exhaustion, depersonalization, and personal accomplishment. All MBI items can be scored from 0 to 6 (7 points), ranging from “never” to “daily.” The MBI has three independent components: emotional exhaustion (EE: 9 items), depersonalization (DP: 5 items), and personal achievement (PA: 8 items). Score ranges define low, moderate, and high levels of each component based on the 0–6 scoring. Cut-off values for the classification of high burnout were based on the European General Practice Research Network (EGPRN) study by Soler et al. (2008) and Rotenstein’s et al. 2018 systematic review of burnout [5]. The authors defined high EE when ≥27 points, high DP when ≥10 points, and low PA when ≤33 points [7].

Demographic and lifestyle items were added to the survey to collect age, gender, graduation year, seniority in the workplace (years of experience), teaching activities (yes/no), work location (urban/semirural), relocation to a hospital during the pandemic (yes/no), being on duty (no/ sometimes /yes), number of leaves of absence among physicians in their primary care centres, having children (yes/no), sleeping well (no/ sometimes /yes), practice sport (no/ sometimes /yes).

### 2.2. Analysis

Data analyses were performed using Stata version 15. Descriptive statistics including mean scores and standard deviations for quantitative variables and frequencies for categorical variables were computed. Burnout mean and prevalence were computed for each time point [5]. 

To assess change in MBI scores over time, were built box plots of burnout score distributions for each domain for times 2 and 3. We also computed the median and interquartile range for each domain. To evaluate the impact of COVID-19 in burnout scores, we used the Wilcoxon matched-pairs signed-rank test for time 2 vs. time 3 with the burnout scales (continuous variable), *z*-value, and *p*-value. We also calculated the difference in the number of individuals with and without burnout (binary variable) and McNemar’s test to calculate chi-squared for the same times. To check the robustness of the results, we calculated the same tests for time 1 vs. time 2. 

To evaluate associations between physician characteristics and burnout, we used multivariate analyses using the GLM procedure for each scale with the outcome variable representing average MBI scores over time for each person. 

## 3. Results

A total of 40 physicians responded to the three surveys; 79% of them were women, with an average age of 47 years (±8 SD), graduated in 1993 (±8.4 SD), with 12 years of experience in the workplace (±7 SD); 90% have children, 80% practice sport, 72.5% responded “yes” or “sometimes” to the question “do you sleep well?”, 52.63% work in urban centres, 10% of them teach, 15% were relocated during the pandemic, 70% were regularly on duty, 50% of them work in centres were at least one or two doctor had taken leave of absence, 57.5% considered changing work place, and 37.5% considered quitting their job altogether (see Table 1). The distribution of the variables age and gender of the sample used matches the distribution of the population studied [18,19]. Additionally, the professionals from the study represent 85% of the primary care centres of the study area. In order to test for interobserver agreement, a group of different physicians from the same area and with the same population demographic were invited to participate in a test. This group and the study participants were given six different scenarios to evaluate regarding type and number of visits, reason for consultation, and other work-related questions. The comparison of answers (using the kappa statistic) from these new physicians with the study physicians showed values ranging from 0.40 to 0.47, which are interpreted as moderate agreement.

Table 2 shows the MBI mean scores and the percentage of high burnout for each domain. We can observe a striking difference between time 1 and 2 for emotional exhaustion, and time 3 for each of the domains. In this table, we can also find the percentage of physicians with high burnout for all domains, which has increased five-fold since the onset of the COVID-19 pandemic.

**Figure 1:**
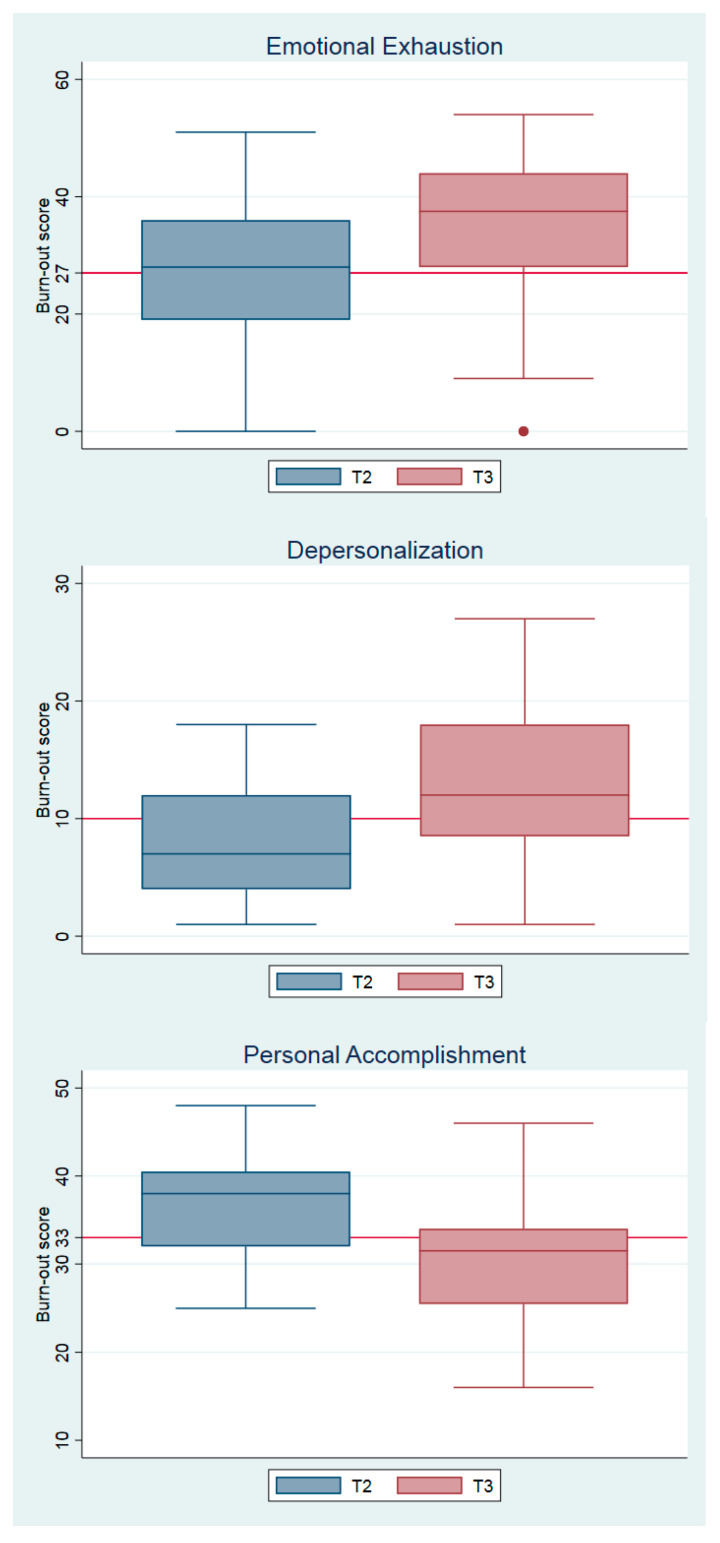
Burnout score for each domain.

Figure 1 shows the burnout score distributions of each domain at time points 2 and 3, with the different cut-off for each domain (cut-off for emotional exhaustion: 27; depersonalization: 10; and professional accomplishment: 33).

The boxplots show a clear worsening for the three domains. For the second point in time (T2: January 2019), emotional exhaustion was just above average, with the other domains below the cut-off. For the third point in time (T3: October 2020), all averages were above the cut-off. The data reflect that in January 2019, 10% of the physicians scored high in all three domains, whereas in October 2020, this percentage soared to 50%.

The Wilcoxon test comparing the data at different points in time showed consistent results. When comparing time 1 vs. time 2, the results do not show statistically significant changes in any of the three domains. In contrast, when comparing time 2 vs. time 3, the changes become significant for emotional exhaustion, depersonalization, and personal accomplishment (see Table 3).

Table 3 also shows that the biggest changes between time 2 and time 3 occur regarding personal accomplishment (*z* = 4.40, *p* = 0.00), with an increase in high burnout of 145% (X^2^ = 0.00). The results also illustrate that emotional exhaustion was already prevalent in 2016 and 2019 but has clearly worsened with the COVID-19 pandemic. Similarly, the pandemic has more than doubled the number of physicians with high burnout in the two remaining domains.

The multivariate model for the average MBI scores revealed significant differences in MBI mean scores by age, having children, and sleep. There were no effects between the other explanatory variables; therefore, the final model is a reduced model with only age, having children, and sleep included as explanatory factors. Age was significant (beta = 0.65 ± 0.26; 95% CI, 0.11 and 1.19; *t* = 2.48; *p* = 0.01): being older increases the chances of having burnout. Having children was also significant (beta = −1.83 ± 6.91; 95% CI, −3.24 and −4.33; *t* = −2.66; *p* = 0.01). The last significant variable was sleep; specifically, individuals not sleeping well scored on average 1.42 points higher than the others (11.83 ± 4.83) (beta = 0.6.13 ± 2.37; 95% CI, 1.31 and 1.09; *t* = 2.58; *p* = 0.01), regarding emotional exhaustion in particular, wherein individuals not sleeping well scored higher (beta = 0.33 ± 0.13; 95% CI, 0.05 and 0.62; *t* = 2.43; *p* = 0.02). When looking specifically at each domain, only sleep was significant for emotional exhaustion, as mentioned. No significant results were found for the other domains regarding the other explanatory variables.

Finally, we also looked at the frequency and characteristics of physicians with no burnout, namely physicians with no high results in at least one of the three domains at T3: there were nine with no emotional exhaustion (22.5%), 13 with no depersonalization (32.5%) and seven with no issues regarding personal accomplishment (17.5%). Of these, the majority were women (66–71%), aged 44 to 49 years, with 12 to 14 years of experience in the workplace; most of them do not teach (87–100%), were not relocated during the pandemic (85–100%), have children (92–100%), and sleep well (76–100%).

## 4. Discussion and Conclusions

Based on the results from the European General Practice Research Network (EGPRN) study by Soler et al. (2008), burnout among primary care physicians is currently at an unprecedented high level. Results obtained prior to the pandemic already indicated a prevalence of burnout of 10 to 20% among primary care physicians [8,9,20,21]. In contrast, burnout levels obtained in this study, conducted during the COVID-19 pandemic, go up to four times such already worrying figures. Whereas the results from the first two periods (2016 and 2019) of the study match EGPRN data, in the third period (October 2020), the number of professionals with high burnout in the three dimensions skyrockets from 10% to 50%.

When analysing trends for all dimensions, we observe an upward trend for emotional exhaustion and depersonalization, and a downward trend for personal accomplishment, even before the onset of the COVID-19 pandemic. Unsurprisingly, all these trends are aggravated with the advance and prolongation of the pandemic, specially depersonalization and personal accomplishment.

The high prevalence of burnout is consistent with findings from a multinational cross-sectional study reporting a burnout prevalence of up to 67% amongst healthcare workers during the pandemic [21]. Different countries such as India [22] and Portugal [23] also report elevated levels of burnout amongst healthcare workers. The study from Portugal, which focuses on primary care workers, reports burnout percentages between 55% and 69%, slightly lower than our 70%. However, the studies use different instruments (the Copenhagen and Oldenburg Burnout Inventories).

Studies exploring the relationship between parenthood and burnout in healthcare professionals are both conflicting and scarce. Whereas Koh et al. [24] asserted that parenthood is a stress risk factor for hospital healthcare professionals, a more recent study conducted during the pandemic indicated that parenthood was related to lower stress levels [25]. Our findings corroborate the role of parenthood as a potential protective factor against burnout among healthcare workers.

The literature on the relationship between burnout and age is also conflicting [26]. In Spain during the COVID-19 pandemic, Torrente et al. [27] found that professionals between 20 and 30 years of age and professionals with more than 15 years of experience were at higher risk of burnout. Further research is needed to elucidate the role of age and time in the profession in burnout [28].

The relationship between suboptimal sleep quality and higher emotional exhaustion that our participants report is consistent with a large number of studies reporting a clear relationship between burnout and disturbed sleep [29]. Indeed, sleep deprivation is amongst the described causes of burnout [30].

As we turn to models exploring the causes of high burnout, such as the Factors Affecting Clinician Well-Being and Resilience put forward by the National Academy of Medicine (NAM), elements such as organizational culture and leadership, staff engagement, scope of practice, and workload stand out as predictors of burnout. Specifically, the following organizational factors have been proven to affect burnout: organization cohesiveness, workload and teamwork, time pressure [11,12,13], and working in chaotic work conditions [13,31]. Most of these factors are deemed to have increased during the pandemic and may partially explain our results.

Similarly, moderators of burnout such as a greater organizational culture [10,31,32], effective team communication [31,33], and staff engagement [34] have diminished during the pandemic.

Physician burnout is associated with negative consequences for the patient and the doctors themselves. It has been linked to lower job satisfaction and higher occupational stress [13], accelerating the number of primary care physicians leaving the practice [13], negative rapport-building [35] and a high turnover in the health services [36].

In Spain, primary healthcare is underfunded and considered unattractive for medical students [37], who tend to choose hospital specialties over general practice, and professionals are already quitting primary care due to burnout [38,39,40]. The current situation, aggravated by the pandemic and also by an increase in workload and an aging population, might become unsustainable in the anticipated and feared scenario of primary care managing the conditions neglected during the pandemic and those arising from it. Despite governments considering budget increases, inherited deleterious organisational dynamics need to be addressed to guarantee adequate patient care.

A limitation of this study is that all respondents were physicians, with no representation of other primary care professionals. We understand that the strategies to improve burnout, reduce stress, and increase the psychological capital of professionals must include all professionals within the organization [41]. Another limitation is the possibility of self-selection bias. In order to enhance the robustness of the analysis, we also checked burnout results for the whole sample of participants in 2019 (*n* = 82) and in 2020 (*n* = 84). Burnout results are similar, with the sample of 40 respondents to the three surveys 1.5% higher and 8% lower than the whole sample for times 2 and 3, respectively.

What are the solutions to burnout? We know that the working environment is largely to blame. The meta-analysis published by Aronsson [42] warned about the great social impact of the demands of the job, the lack of control of the environment, and lack of support. For health professionals, the organisational factor is extremely relevant. The results of this meta-analysis corroborate the need to improve the working environment to prevent burnout.

In our health area, a support strategy was put in place specifically for health professionals affected by the pandemic [43]. An evaluation of this strategy conducted by our group using mixed methods (accepted for publication) showed intermediate levels of satisfaction (6.54 on a scale of 0 to 10); the lowest scores were given to psychological support. We believe that the support should concomitantly address biological, psychological, and social issues, since the strategy fails when any of these three elements is missing.

Mid- and long term, experts emphasize the need to strengthen primary care, update the management, and provide the resources to transform primary care into the cornerstone of healthcare services. Heavy investment is required, since an analysis by the OECD in the majority of its member states for the 2000–2017 period indicates that the proportion of family doctors have been progressively decreasing.

In 2019, the group of primary care professionals FoCAP (https://focap.wordpress.com/ accessed on 17 August 2021) launched a manifesto [44] with specific actions to reduce burnout, such as changing organizational factors (improving how organizations treat their professionals, modernizing leadership and team management), structural factors (relationship with specialized and hospital care, teaming with nurses, reducing paperwork), work factors (change of payment model and productivity incentives, more flexible work, and work-family conciliation) and professional development (continuing training and research).

During the devastating effect of the SARS-Cov-2 pandemic, professional associations and scientific societies proposed in 2020 a decalogue of measures to improve the work environment for health professionals [45]:

1. A new agreement for health among political forces in Catalonia and Spain;
2. More economic resources: an estimated 5 billion euros of additional funding for health in Catalonia and of 25 billion in Spain;
3. Better conditions and resources for health professionals, using 60% of the additional funding. Emphasis on increased salaries for junior staff and staff in training;
4. Better facilities and infrastructure: 40% of additional funding to replace obsolete resources;
5. Professionalism: the reform must be based on the values, knowledge, and experience of healthcare professionals;
6. Health organisation: participative definition of healthcare goals and challenges of the Catalonia Health Plan. Results-based; autonomy in management; flexibility of working models;
7. Coordination and patient-centered care across the various healthcare services;
8. Greater social emphasis. Linking social and health services, with a new support model for the elderly;
9. A solid public health service ready for the new challenges. Protection, prevention, participation. International coordination;
10. The experience of COVID-19, an opportunity to learn from failures and to strenghthen assets. “Without health, there is no future”.

In brief, measures aimed to improve the current situation of health professionals should:

- Establish a new social and political agreement to provide much needed additional resources;
- Transform organizational models of primary care centres, making them more adaptable (improving leadership and decision-making) and improving their coordination with specialists and hospitals;
- Empower primary care professionals with more self-management, training, and opportunities;
- Reduce paperwork;
- Promote and develop the values of primary healthcare [46].

In conclusion, burnout is reaching alarming proportions among family doctors since the onset of the COVID-19 pandemic. However, COVID-19 has only aggravated and exposed structural and organizational dimensions of the primary care services in Catalonia unable to cope with demands that are only expected to increase, irrespective of pandemics. Innovative solutions are urgently needed to avoid a primary healthcare crisis.

## Figures and Tables

**Table 1:** Descriptive statistics.

Variable	Obs.	Mean (%)	Std. Dev.	Min	Max
**Socio-demographic factors**					
Age	39	47.44	8.00	32	63
Gender					
Female	31	79.49			
Male	8	20.51			
Graduation year	39	1993	8.40	1977	2012
Seniority	38	12.42	7.04	1	29
Have children					
No	6	15			
Yes	34	85			
Do you practice sport?					
No	8	20			
Sometimes	8	20			
Yes	24	60			
Do you sleep well?					
No	11	27.5			
Sometimes	6	15			
Yes	23	57.5			
**Work-related factors**					
Work location					
Urban	18	47.37			
Semirural	20	52.63			
Are they teaching?					
No	35	89.74			
Yes	4	10.26			
Being on duty					
No, almost never	12	30			
Sometimes	10	25			
Regularly	18	45			
Relocation during pandemic					
No	4	10			
Yes	36	90			
How many doctors have taken leave of absence in your centre?					
0	11	27.5			
1	10	25			
2	11	27.5			
3	5	12.5			
4	2	5			
8	1	2.5			
Have you thought about changing your work place?					
No	17	42.5			
Yes, in Spain	20	50			
Yes, outside Spain	3	7.5			
In the lasts months, have you thought about quitting the profession?					
No	25	62.5			
Yes	15	37.5			

**Table 2:** MBI mean scores, prevalence.

	MBI Scores	Mean ± SD *	% High Burnout	% with 3 Domains High
Time 1	Emotional Exhaustion	227 ± 12.6	37.5%	7.5%
	Depersonalization	7.9 ± 7.2	32.5%	
	Personal Accomplishment	37.1 ± 7.2	27.5%	
Time 2	Emotional Exhaustion	26.4 ± 122	55.0%	10%
	Depersonalization	7.8 ± 5.1	30.0%	
	Personal Accomplishment	36.8 ± 6.3	27.5%	
Time 3	Emotional Exhaustion	35 ± 13.2	77.5%	50%
	Depersonalization	13.2 ± 6.5	70.0%	
	Personal Accomplishment	30.4 ± 7.3	67.5%	

Abbreviations: MBI: Maslach Burnout Inventory. * *p* ≤ 0.05.

**Table 3:** Stability MBI scores (change).

	T1	T2	T3	T2 vs. T3				T1 vs. T2			
	Median (IQR)	Median (IQR)	Median (IQR)	*z* Value	*p* Value	No. (%)	X^2^	*z* Value	*p* Value	No. (%)	X^2^
Emotional Exhaustion	23 (19)	28 (17)	37.5 (16)	3.87	0.00	9 (40)	0.00	1.81	0.06	7 (46)	0.07
Depersonalization	5.5 (9)	7 (8)	38 (8.5)	4.29	0.00	16 (133)	0.00	−0.26	0.79	−1 (7)	0.73
Personal Accomplishment	38 (9)	38 (7.7)	31.5 (8.5)	4.40	0.00	16 (145)	0.00	0.22	0.82	0	1.00

Abbreviations: T: time, IQR: interquartile range.

## Data Availability

Data available on request due to restrictions.
